# Late cardiotoxicity related to HER2-targeted cancer therapy

**DOI:** 10.1186/s40959-024-00215-3

**Published:** 2024-03-08

**Authors:** Isabelle Senechal, Maria Sol Andres, Jieli Tong, Ylenia Perone, Sivatharshini Ramalingam, Muhummad Sohaib Nazir, Stuart D Rosen, Nicholas Turner, Alistair Ring, Alexander R Lyon

**Affiliations:** 1 Cardio-Oncology Service, Royal Brompton Hospital, Guy’s and St. Thomas’ NHS Foundation Trust, London, UK; 2 Centre hospitalier universitaire de Québec, Québec, Canada; 3 Royal Marsden Hospital Foundation Trust, London, UK; 4 School of Biomedical Engineering and Imaging Sciences, Faculty of Life Sciences and Medicine, King’s College London, London, UK; 5 National Heart and Lung Institute, Imperial College London, London, UK

**Keywords:** Breast cancer, Cardio-oncology, Cardiotoxicity, Left ventricular dysfunction, HER2-targeted therapy

## Abstract

Long-term anti-HER2 therapy in metastatic HER2 + cancers is increasing, but data about the incidence and risk factors for developing late Cancer therapy-related cardiac dysfunction (CTRCD) are missing. We conducted a single-centre, retrospective analysis of a cohort of late anti-HER2 related cardiac dysfunction referred to our Cardio-Oncology service. We include seventeen patients with metastatic disease who developed CTRCD after at least five years of continuous anti-HER2 therapy. Events occurred after a median time of 6.5 years (IQR 5.3-9.0) on anti-HER2 therapy. The lowest (median) LVEF and GLS were 49% (IQR 45–55) and − 15.4% (IQR − 14.9 - -16.3) respectively. All our patients continued or restarted, after a brief interruption, their anti-HER2 therapy. Most (16/17) were started on heart failure medical therapy and normalized their left ventricular ejection fraction at a follow-up. Our study has demonstrated that CTRCD can occur after many years of stability on anti-HER2 therapy and reinforces the importance of continuing cardiovascular surveillance in this population.

Human Epidermal Growth Factor Receptor-2 (HER-2) targeted therapies have revolutionized the treatment of HER2 + breast cancer and are the current standard of care for adjuvant therapy in early stages, and for long-term therapy in recurrent and metastatic HER2 + disease [1]. Cancer therapy-related cardiac dysfunction (CTRCD) is a recognised complication of these treatments, and it usually presents as an early, asymptomatic decline in left ventricular ejection fraction (LVEF) [1] in the first 12 months of treatment. In recent years, with the increasing use of long-term HER2-targeted therapy in the metastatic setting, some cases of late left ventricular systolic dysfunction (LVSD) have been described [2, 3]. However, data are limited and the risk factors for developing cardiac toxicity after several years of treatment are not known. To our knowledge, this is the first report describing a population with late cardiotoxicity after at least five years of continuous anti-HER2 therapy.

We conducted a single-centre, retrospective analysis of a cohort of late anti-HER2 therapy-related cardiac dysfunction referred to the Cardio-Oncology service at Royal Brompton Hospital from 2011 to 2023. We defined ‘late’ as the occurrence of reduction in LVEF at a time point following at least 5 years of continuous anti-HER2 therapy. We used the International Cardio-Oncology Society (ICOS) definitions of CTRCD to categorize severity [4]. As our database included patients referred before 2022, we also included cases meeting the previous definitions of anti-HER2-induced LV systolic dysfunction, namely a ≥10% reduction in LVEF and/or to below institutional lower limit of normal [5].

We identified seventeen patients, referred to our clinic between 2018 and 2023, fulfilling our inclusion criteria. Table 1 summarizes patients’ demographic. Our cohort included sixteen women (94%) with metastatic HER2 + breast cancer and one man with metastatic HER2 + gastric adenocarcinoma. The median age at the time of CTRCD diagnosis was 58 years (IQR 54–61). The majority of patients were on long-term 3 weekly Trastuzumab monotherapy (59%) or on long-term 3 weekly Trastuzumab and Pertuzumab (29%). All patients had a normal baseline echocardiogram with a mean LVEF of 63.7 ± 6.0%. Four patients (24%) had hypertension, two patients (12%) had diabetes, one patient (6%) was an ex-smoker, and one patient (6%) was obese. No patients had clinically significant preexisting heart disease (heart failure, coronary artery disease and significant valvular disease). Nearly half of our patients (47%) received anthracycline chemotherapy prior to their anti-HER2 therapy, and 5 patients (29%) had previous left chest radiotherapy. At the time of the CTRCD diagnosis, none were receiving other cancer treatments with potential cardiotoxic effects. Two patients (12%) had a prior history of early anti-HER2 toxicity. According to the HFA-ICOS baseline risk calculator for anti-HER2 therapies, 7 patients (41%) were at low risk, 7 patients (41%) were at medium risk and 3 patients (18%) were at high risk of CTRCD.

**Table 1:** Demographics

	N (n total = 17)
Age at referral (median)	58 (IQR 54–61)
Age at cancer diagnose (median)	50 (IQR 46–55)
Women (%)	16 (94%)
Breast cancer (%)	16 (94%)
Gastric cancer (%)	1 (6%)
Baseline LVEF (mean)	63.7 ± 6.0%
Type of anti-HER2 therapy (%)	
Trastuzumab only	10 (59%)
Pertuzumab + Trastuzumab	5 (29%)
TDM1	1 (6%)
Tucanitib + Trastuzumab	1 (6%)
Concomitant systemic treatment	
Capecitabine	2 (12%)
Tamoxifen	2 (12%)
Denosumab	4 (24%)
Cardiovascular risk factor (%)	
Hypertension	5 (29%)
Diabetes	2 (12%)
Smoker/ex-smoker	1 (6%)
Obesity	1 (6%)
Prior anthracyclines exposure (%)	8 (47%)
Prior left chest radiotherapy (%)	5 (29%)
Other previous chemotherapy	
Taxanes	13 (76%)
Fluoropyridines	4 (24%)
Cyclophosphamide	1 (6%)
Mitoxantrone	1 (6%)

The panel A of the figure represents the distribution of CTRCD severity. Nine patients (53%) were diagnosed with asymptomatic mild CTRCD, 5 patients (29%) with asymptomatic moderate CTRCD, 1 patient (6%) with symptomatic moderate CTRCD and 2 patients (12%) with symptomatic severe CTRCD. Events occurred after a median time of 6.5 years (IQR 5.3-9.0) on anti-HER2 therapy. The median for the lowest LVEF and GLS were 49% (IQR 45–55) and − 15.4% (IQR − 14.9 - -16.3) respectively. Sixteen patients (94%) were started on heart failure therapy, mainly with ACEi/ARB (88%) and/or beta blockers (65%), in line with the guidelines, with carvedilol the preferred betablocker.

**Fig. 1:**
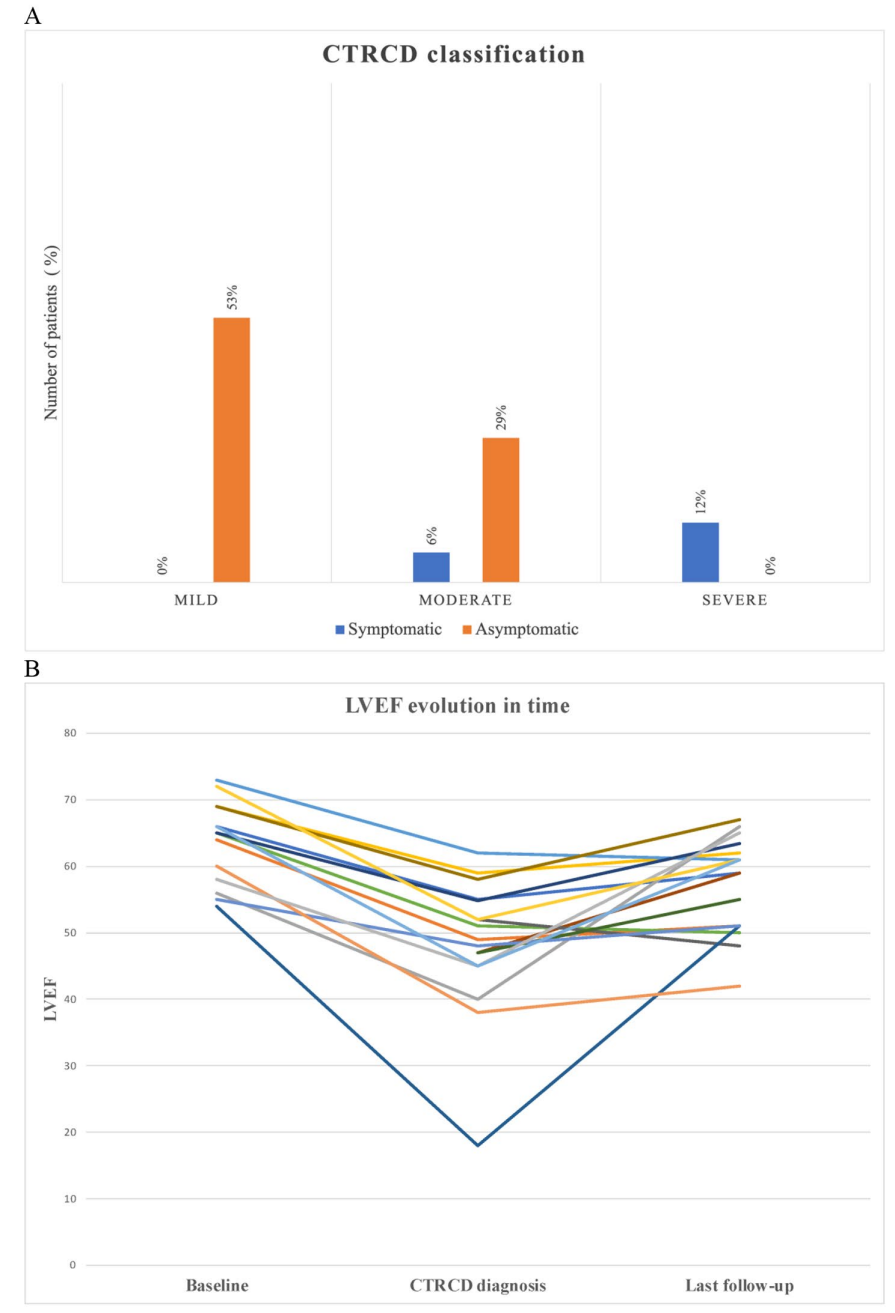
**A**. CTRCD severity classification. **B**. LVEF evolution

Ten patients (59%) continued their anti-HER2 therapy uninterrupted. Seven patients (41%) had their anti-HER2 therapy temporarily interrupted for a median duration of 3 months (IQR 1.5–3.3), before restarting anti-HER2 therapy in all cases. One patient experienced cancer progression during their treatment interruption.

Panel B presents the LVEF evolution for each patient during follow-up. The majority of the cohort had complete (11/17 = 65%) or partial (3/17 = 18%) recovery of their systolic function. Median LVEF at last follow-up was 59% (IQR 51–62) whilst continuing anti-HER2 therapy. The recovery of left ventricle (LV) systolic function was rapid and occurred within a median of 2 months (IQR 1.5–2.9). We identified the presence of a second cardiac insult, contributing to the decline in LVEF in nine cases (53%). This second hit was COVID-19 infection with COVID-19-related myocarditis for 4 patients (24%), COVID-19 vaccine-related myocarditis for one patient (6%), change of anti-HER2 agent for 2 patients (12%), and supra-ventricular arrhythmia for two patients (12%).

CTRCD can occur after many years of stability on anti-HER2 therapy, even in patients with a low baseline risk. Our analysis suggests that a second hit increases the risk of developing LVSD. A previous case report described late LVEF decline following the addition of Pertuzumab to ongoing long-term Trastuzumab [3]. We observed the same phenomenon, with a change of the anti-HER2 agent as the second hit for two patients. Both of our patients were switched from trastuzumab to trastuzumab deruxtecan (Enhertu). Half of our patients did not have any concomitant event to explain the decline in LVEF which may reflect the cumulative effect of anti-HER2 targeted therapy. All the cohort had normal baseline echocardiogram with no history of previous cardiac diseases, and the majority did not have any cardiovascular risk factors. These data suggest a causality between anti-HER2 therapy and new LVSD. Some of our patients received anthracyclines a couple of years before their diagnosis of CTRCD, and it could have contributed to the LVEF decline. According to our data, late anti-HER2-related cardiac dysfunction remains a limited and reversible condition when managed properly. All our patients were able to continue their anti-HER2 therapy with the introduction of cardioprotective therapy, and the majority normalized their LV systolic function. LVEF recovery was observed in less than 3 months, which corresponded mainly to the interval between CTRCD diagnosis and the repeat imaging. These findings are consistent with the current literature showing that anti-HER2 induced LVSD is a reversible phenomenon [6] and does not prevent the continuation of the treatment under cardioprotective medication [7, 8].

Our study is limited by its retrospective nature and small sample size. Unfortunately, cardiac biomarkers, including troponin and brain natriuretic peptide, at CTRCD diagnosis were not available in most cases. GLS values at diagnosis were also missing for almost half of our cohort, which may be related to the technical challenges of undertaking GLS measurements in breast cancer patients. The exact baseline LVEF was unavailable for one of our patients, even thought it was reported as normal in her chart. Another important limitation of our study is that we did not have access to the total number of patients on continuous anti-HER2 therapies for at least 5 years. This precludes an estimation of the frequency of the late CTRCD induced by anti-HER2 therapies. Therefore, our study can only be interpreted as a descriptive analysis.

Further studies are needed to define the subpopulation at risk of developing very late cardiotoxicity, perhaps through multi-centre registries or novel imaging biomarkers. Our study reinforces the importance of continuing regular clinical cardiovascular surveillance for patients undergoing long-term anti-HER2 therapies, especially as CTRCD is often asymptomatic. The ESC Guidelines [8] recommends 6-monthly echocardiograms in the setting of metastatic HER2 + cancers on long-term therapy. This provides the opportunity to detect early stages of CRTCD, initiate cardioprotective therapy, and most importantly allow the continuation of highly effective anti-HER2 therapy uninterrupted in a patient population where the absolute benefit of the anti-HER2 therapy is high.

## Data Availability

No datasets were generated or analysed during the current study.

## Abbreviations

HER2: Human Epidermal Growth Factor Receptor-2
CTRCD: Cancer therapy related cardiac dysfunction
LVEF: Left ventricle ejection fraction
ICOS: International Cardio-Oncology Society
IQR: Interquartile range
GLS: Global longitudinal strain
LV: Left ventricle
LVSD: Left ventricular systolic dysfunction
